# Factors That Motivate Older Adults to Participate in Research: Typologies and Implications

**DOI:** 10.1093/geroni/igab046.2118

**Published:** 2021-12-17

**Authors:** Dawn Carr, Zhe He, Mia Lustria, Shubo Tian, Maedeh Agharazidermani, Walter Boot

**Affiliations:** 1 Florida State University, Tallahassee, Florida, United States; 2 School Of Information, Florida State University, Florida, United States

## Abstract

A key challenge for scholars who study aging is identifying a pool of research volunteers willing to participate. Toolkits and strategies acknowledge the differences in recruitment needed for older adults relative to younger adults, but there is little information about variations among older adult research volunteers. Based on a community sample of older adults age 60+, this study evaluates differences across seven specific motivators across three broad categories: values/altruism, personal growth/improvement, and immediate gratification. We then identify and evaluate four typologies of older adult volunteers based on the combinations of motivations the older adults in our sample identify as important to participation in research studies. Based on these analyses, we describe how our results might inform recruitment and retention practices in aging studies. Further, we will discuss how these results will help shape our technology-based reminder system with a greater understanding of motivations.

